# Atypical presentation of syphilis: Rapidly progressive glomerulonephritis

**DOI:** 10.1002/ccr3.6864

**Published:** 2023-01-16

**Authors:** Semir Abdi Usmael, Tesfay Atsbaha Gebremedhin

**Affiliations:** ^1^ Internal Medicine Department College of Health and Medical Sciences, Haramaya University Harar Ethiopia

**Keywords:** case report, Ethiopia, hematuria, rapidly progressive glomerulonephritis, syphilis

## Abstract

Syphilis is a sexually transmitted disease with a wide range of clinical manifestations. With the recent worldwide resurgence of syphilis, it is imperative to recognize various presentations of this great imitator. Renal syphilis is rare and most commonly present as nephrotic range proteinuria associated with pathological features of membranous glomerulonephritis. Rapidly progressive glomerulonephritis (RPGN) is a rare and atypical form of renal syphilis. A 50‐year‐old Ethiopian woman presented with periorbital swelling, hematuria, proteinuria, and rapidly progressive renal failure. Rapid plasma reagin and confirmatory Treponema pallidum hemagglutination (TPHA) tests were reactive. Treatment with a weekly Benzathine penicillin for 3 weeks resulted in a rapid return of renal function to baseline, with the increasing rate of new syphilis, clinicians should be mindful of the various renal manifestation of syphilis. This case highlights the significance of considering syphilis as a reversible cause in any patient presenting with a clinical feature suggestive of RPGN.

## 1 BACKGROUND

Syphilis is a multisystemic infectious disease with various presentations hence the designation “great imitator.” Even though renal involvement is rare, there are increasing reports of various renal lesions due to the recent global resurgence of syphilis. Kidney involvement can occur during the primary, latent, and tertiary stages of syphilis.^1^ It can affect every structure of the kidney resulting in glomerulopathies, tubular pathology, and vasculitic lesions in the kidney.^1^, ^2^, ^3^, ^4^, ^5^, ^6^, ^7^, ^8^, ^9^, ^10^, ^11^, ^12^, ^13^, ^14^, ^15^, ^16^, ^17^, ^18^, ^19^

Various types of glomerulonephritis have been described.^6^ The most common renal manifestation of syphilis is membranous nephropathy, presenting with acute onset of nephrotic syndrome or nephrotic range proteinuria and this may be associated with features suggestive of secondary syphilis such as maculopapular rash, mucocutaneous lesion, and lymphadenopathy.^12^, ^13^, ^14^, ^15^ Other syphilis‐associated glomerular diseases are minimal change disease (MCD), focal segmental glomerulosclerosis (FSGS), and membranoproliferative glomerulonephritis (MPGN).^11^, ^16^, ^17^

On the other hand, the rapidly progressive glomerulonephritis (RPGN) pattern is an atypical and rare presentation of syphilis, with only three case reports.^2^, ^3^, ^4^ We report a case of an immunocompetent patient presenting with hematuria, proteinuria, and rapidly deteriorating renal function suggestive of RPGN. This case also emphasizes challenges in the diagnosis and management of RPGN in resource‐limited settings.

## 2 CASE PRESENTATION

A 50‐year‐old Ethiopian woman presented with a 2‐week history of facial swelling and reddish discoloration of urine. She was in her usual state of health until 2‐week before admission, when she developed nonprojectile vomiting of ingested matter and facial swelling that progressed to involve both legs. She also complained of reddish discoloration of urine, decreased urine volume, dull‐aching left flank pain with no radiation, anorexia, and fatigue. She also provided a history of shortness of breath on exertion and intermittent dry cough. Otherwise, she denied hemoptysis, nasal discharge, epistaxis, skin rash, joint pain, atopy, asthma, fever, night sweat, and other systemic symptoms. She did not have preceding diarrhea, gastrointestinal bleeding, sore throat, orthopnea, PND, visual disturbance, headache, neck pain, neck stiffness, and change in mentation. past medical history was nonrevealing. Similarly, her sexual history was also unremarkable. She has never been diagnosed or treated for syphilis. She is not on any medication; she did not smoke tobacco, use illicit drugs, or drink alcohol. Her family history was unremarkable.

On examination, her vital signs were as follows blood pressure of 190/100 mmHg, pulse rate of 77 beats per minute, respiratory rate of 20 breaths per minute, temperature of 36.8°C, and oxygen saturation of 98% while breathing ambient air. There was periorbital swelling and grade one symmetric bilateral pitting edema of the legs. A cardiac examination revealed a grade 2 diastolic murmur heard at the aortic area, but jugular venous pressure (JVP) was not raised and there was no pericardial rub. There was no sinus tenderness, nasal mucosal ulceration, septal deviation, skin rash, lymphadenopathy, genital ulcer, and joint swelling or tenderness. Chest and neurologic examinations were nonrevealing.

Laboratory workup disclosed Serum creatinine of 9.28 mg/dl, blood urea nitrogen 145 mg/dl, and hemoglobin of 10.3 mg/dl with MCV of 79.8 fl (Table 1). Urinalysis was positive for +2 albumin and +3 blood. On urine microscopy, there were 10–15 RBC/HPF and 0–4 WBC/HPF. Rapid plasma reagin and confirmatory TPHA reactive. Other serologic tests like antinuclear antibody (ANA), antinuclear cytoplasmic antibody (ANCA), antiglomerular basement membrane antibody (anti‐GBM), and complement level were not done because of financial reasons and the unavailability of laboratory settings in the region. Abdominopelvic ultrasound showed normal‐sized kidneys with increased parenchymal echogenicity and mild ascites. Chest radiography revealed minimal bilateral pleural effusion and increased cardiothoracic ratio. Transthoracic echocardiography showed thickening of the aortic valve with mild aortic regurgitation but no signs of vegetation and intracardiac abscess. A renal biopsy was not done due to a lack of service in our institution.

**TABLE 1:** Laboratory data on admission

Variables	Reference range	On admission (28/3/2021)
White cell count (per μl)	4000–10,000	6260
Differential cell count (per μl)		
Neutrophile	2000–7000	3750
Lymphocyte	600–4100	2030
Eosinophile	0–500	187
Hemoglobin (mg/dl)	11–16.5	10.3
Hematocrit (%)	35–56	31.7
MCV (fl)	80–99	79.8
MCH (pg)	26.5–33.5	26
MCHC (g/L)	32–36	32.6
RDW (%)	10–15	12.9
Platelet count (per μl)	100–300	276
Creatinine (mg/dl)	0.5–1.2	9.28
Blood urea nitrogen (mg/dl)	7–20	145
Sodium (mmol/L)	136–146	136
Potassium (mmol/L)	3.5–5	4.9
Chloride (mmol/L)	102–109	120.4
AST (IU/L)	13–28	21
ALT (IU/L)	7–41	13
ALP (IU/L)	60–360	173
Total bilirubin (mg/dl)	<1.2	0.6
Direct bilirubin (mg/dl)	<0.4	0.2
Albumin (mg/dl)	3.5–5.5	3.59
Random blood glucose (mg/dl)		86
Lipid panel		
Total cholesterol (mg/dl)	<200	194
Triglyceride (mg/dl)	<200	35
HDL (mg/dl)	>40	56
LDL (mg/dl)	<160	131
Uric acid (mg/dl)	2.5–7	9.9
Urinalysis		
Specific gravity		1.010
pH		6.6
Albumin		+2
Blood		+3
WBC		0–4
RBC		10–15
Cast		–
24‐h urine protein		N/A
UPCR/UACR		N/A
Serology		
Hepatitis B		Negative
Hepatitis C		Negative
HIV		Negative
RPR		Positive
TPHA		Positive
Anti‐ASO antibody		Negative
ANA		N/A
ANCA		N/A
Anti‐GBM antibody		N/A
Complement (C3 and C4)		N/A
CRP (mg/L)		N/A
ESR (mm/h)		N/A
RF		N/A

Abbreviations: ALP, alkaline phosphatase; ALT, alanine transaminase; ANA, antinuclear antibodies; ANCA, antinuetrophilic anticytoplasmic antibody; anti‐GBM, antiglomerular basement membrane; AST, aspartate transaminase; CRP, C reactive protein; ESR, erythrocyte sedimentation rate; HIV, human immunodeficiency virus; N/A, not available; RBC, red blood cell; RF, rheumatoid factor; RPR, rapid plasma reagin test; TPHA, treponema hemaglutination; WBC, white blood cell.

With a working diagnosis of syphilis‐related rapid glomerulonephritis, the patient was admitted and started on intramuscular benzathine penicillin G 2.4 million IU weekly for three doses, intravenous furosemide 40 mg twice daily, amlodipine 10 mg once daily by mouth, intravenous metoclopramide 10 mg thrice daily, intravenous omeprazole 20 mg once daily, and no salt‐added diet. She was also challenged with 500 ml of normal saline for possible superimposed prerenal component due to vomiting. She did not receive steroids or any other form of immunosuppressive therapy. Two days after, the first dose of benzathine penicillin G, the serum creatinine declined rapidly from 9.28 to 7 mg/dl. After the third dose, it became 0.98 mg/dl and had been stable since (Table 2). Currently, she feels well, nonedematous, normotensive, and has a stable renal function.

**TABLE 2:** Timeline of the patient's serial laboratory investigation

Variables	Admission (Day 0)	29/3/21	31/3/21	5/4/21 (2‐day posttreatment)	23/5/21 (2‐week posttreatment)	22/6/21 (6‐week posttreatment)	22/7/21 (10‐week posttreatment)
Creatinine (mg/dl)	9.28	9.7	9.03	7	1.2	0.98	0.7
Urinalysis							
Albumin	+2				+2	+2	+1
Blood	+3				+3	+2	+1
RBC cast	10–15/HPF				Many	Many	0–2

## 3 DISCUSSION

We present a case of syphilis‐related RPGN with a dramatic and sustained response to a course of penicillin therapy. This case implicates the importance of considering syphilis as a reversible cause in any patient presenting with RPGN.

Syphilis is a sexually transmitted infection caused by spirochaete Treponema pallidum. It presents a wide range of symptoms and signs depending on the stage of the disease. Kidney involvement is rare with prevalence ranging from 0.3% to 10% in patients diagnosed with secondary syphilis.^5^

RPGN is an atypical and very rare presentation of syphilis. Similar to our case, in three previously reported cases patient presented with hematuria, proteinuria, and rapidly increasing serum creatinine over a short period.^2^, ^3^, ^4^ There may or may not be associated features suggestive of secondary syphilis.

The pathogenesis consists of immune complex‐mediated glomerular injury as evidenced by the presence of treponemal antigens and antibodies to *Treponemal pallidum* on pathologic examination of renal tissue.^7^ Syphilis‐related glomerulonephritis is diagnosed based on positive serology, histologic evidence of GN with rapid and sustained resolution after treatment with penicillin, or the presence of antitreponemal antibodies or treponemal antigens in the glomerular deposits.^8^

Treatment of syphilis‐related RPGN includes parenteral penicillin for underlying syphilitic infection.^9^, ^10^ Similar to the present case, Walker et al. and Tognetti et al. reported complete and rapid resolution of renal lesions and normalization of renal function with penicillin alone.^2^, ^11^ The role of immunosuppressive therapy is unknown, given the paucity of evidence on whether it is beneficial for induction in the acute treatment phase. Qi et al. treated a syphilitic patient with crescentic necrotizing glomerulonephritis with a 14‐day course of penicillin and a short course of high‐dose penicillin which resulted in a dramatic response. However, such a response to a short course of steroids is not consistent with ANCA‐associated RPGN and suggests the response is due to penicillin.^4^

Our patient presented with the typical clinical feature of RPGN; active urine sediment with rapidly deteriorating renal function. A positive confirmatory treponemal test along with rapid and sustained improvement in renal function after a course of penicillin supports the diagnosis of syphilis‐related RPGN.

Although guidelines for the diagnosis and treatment of RPGN exist, it is infrequently implemented in resource‐limited settings. Lack of capacity to undertake and evaluate kidney biopsy, unavailability of ancillary serologic tests, lack of trained nephrologists and nephropathologist, and lack of access to immunosuppressive agents and renal replacement therapy are some of the barriers to implementing this evidence‐based guideline.^20^ Clinicians in resource‐limited settings diagnose RPGN mostly based on clinical ground and locally available ancillary serologic tests. Despite this limitation in accurate diagnosis, a significant proportion ends up being treated empirically with an immunosuppressive agent. Even though this is undertaken with good intent, such a strategy can be detrimental to patient health. Since the glomerular disease is one of the few reversible causes of renal failure in a resource‐limited setting, international cooperation and due attention is required to improve the outcome of these patients.

There were several limitations in the management of this case, namely the lack of renal biopsy to confirm RPGN and the lack of serologic tests to rule out other causes of RPGN.

## 4 CONCLUSIONS

RPGN is a very rare and atypical presentation of syphilis. However, with a global reemergence of syphilis, it is essential to recognize this atypical renal manifestation of syphilis. This case highlights the significance of considering syphilis as a reversible cause in any patient presenting with a clinical picture suggestive of RPGN.

## AUTHOR CONTRIBUTIONS

**Semir Abdi Usmael:** Conceptualization; data curation; formal analysis; investigation; methodology; resources; software; validation; writing – original draft; writing – review and editing. **Tesfay Atsbaha Gebremedhin:** Resources; validation; writing – original draft; writing – review and editing.

## FUNDING INFORMATION

None.

## CONFLICT OF INTEREST

The authors declare that they have no conflict of interest.

## DATA AVAILABILITYSTATEMENT

Not applicable.

## ETHICS STATEMENT

Not applicable.

## CONSENT

Written informed consent was obtained from the patient to publish this report in accordance with the journal's patient consent policy. A copy of written consent is available for review by editor‐in‐chief of this journal.
